# Long-Term Effect of Agricultural Reclamation on Soil Chemical Properties of a Coastal Saline Marsh in Bohai Rim, Northern China

**DOI:** 10.1371/journal.pone.0093727

**Published:** 2014-04-02

**Authors:** Yidong Wang, Zhong-Liang Wang, Xiaoping Feng, Changcheng Guo, Qing Chen

**Affiliations:** 1 Tianjin Key Laboratory of Water Resources and Environment, Tianjin Normal University, Tianjin, China; 2 State Key Laboratory of Environmental Geochemistry, Institute of Geochemistry, Chinese Academy of Sciences, Guiyang, China; 3 College of Urban and Environmental Sciences, Tianjin Normal University, Tianjin, China; Institute of Genetics and Developmental Biology, Chinese Academy of Sciences, China

## Abstract

Over the past six decades, coastal wetlands in China have experienced rapid and extensive agricultural reclamation. In the context of saline conditions, long-term effect of cultivation after reclamation on soil chemical properties has not been well understood. We studied this issue using a case of approximately 60-years cultivation of a coastal saline marsh in Bohai Rim, northern China. The results showed that long-term reclamation significantly decreased soil organic carbon (SOC) (−42.2%) and total nitrogen (TN) (−25.8%) at surface layer (0–30 cm) as well as their stratification ratios (SRs) (0–5 cm:50–70 cm and 5–10 cm:50–70 cm). However, there was no significant change in total phosphorus (TP) as well as its SRs under cultivation. Cultivation markedly reduced ratios of SOC to TN, SOC to TP and TN to TP at surface layer (0–30 cm) and their SRs (0–5 cm:50–70 cm). After cultivation, electrical conductivity and salinity significantly decreased by 60.1% and 55.3% at 0–100 cm layer, respectively, suggesting a great desalinization. In contrast, soil pH at 20–70 cm horizons notably increased as an effect of reclamation. Cultivation also changed compositions of cations at 0–10 cm layer and anions at 5–100 cm layer, mainly decreasing the proportion of Na^+^, Cl^−^ and SO_4_^2−^. Furthermore, cultivation significantly reduced the sodium adsorption ratio and exchangeable sodium percentage in plow-layer (0–20 cm) but not residual sodium carbonate, suggesting a reduction in sodium harm.

## Introduction

Coastal wetlands provide essential ecosystem services to people and environment including flood protection, water supply and purification, food productivity, erosion control, wave attenuation, shoreline stabilization, wildlife habitat, biodiversity, climate regulation and amenity [1], [2]. Over the past century, natural coastal wetlands all over the world have been rapidly shrunk due to intensive anthropogenic activities [3], [4], [5], [6]. In China, approximately 51% (2.2×10^4^ km^2^) of coastal natural wetlands were lost or degraded since the 1950s, primarily due to agricultural reclamation [7]. Soil is one of the foundations of ecosystem services of wetland and cropland. Therefore, it is important to study the influence of agricultural cultivation after reclamation on soil properties of the coastal wetlands.

Some studies have evaluated the reclamation-induced changes of soil properties in coastal wetlands. Conversion of coastal wetlands to croplands has been reported to cause radical changes in soil chemical properties because of alterations of hydrology and agricultural activities. First, hydrologic alterations such as ditch drainage and diking led to increase in aeration and decrease in salinity [8], [9], [10], [11]. Aeration accelerated soil organic matter decay [4], [12], [13] and affected substances' characteristics [5] and mobility through changing redox conditions [8], [14]. Changes in salinity further influenced substances' cycle in soils [15], [16]. Second, application of fertilizer affected nutrient contents such as carbon [17], nitrogen and phosphorus [14], [18]. In addition, agricultural managements including planting, harvesting and tillage also influenced balances of substances inputs and outputs in soils [19], [20], [21]. Most of these studies were focusing on short-term scales (e.g. [5], [8], [11], [13], [14]). In contrast, only few studies were available at long-term scales [4], [9], [10].

Stratification of soil chemical properties with soil depth is common in many natural ecosystems [4], [22]. Different soil horizons of coastal wetlands may have distinct responses to agricultural reclamation. The surface soil is the vital interface that receives intense impact from human disturbance. In support of this concept, chemical characteristics of the surface soil in coastal wetlands were demonstrated to be more sensitive to agricultural reclamation [4], [18]. However, quantitative expression of the horizon-induced differences in response of coastal wetland to agricultural reclamation is rare. Stratification ratio (SR) is a quantitative indicator to represent changes of soil profile feature [22]. It is hypothesized that the SR can be used as an indicator of the responses of different soil horizons to human disturbance.

In Bohai Rim of northern China, coastal marsh geogenesises have been realized naturally by land-sea interactions since the middle-late Holocene. Since the founding of New China, natural coastal marshes have been largely reclaimed for agriculture to alleviate the pressure of the increasing population. For instance, approximately 55% of coastal natural wetlands in Tianjin were disappeared since the 1950s. However, it is unclear that how agricultural reclamation affects soil chemical properties and their SRs of the coastal saline marshes in Bohai Rim, northern China.

The objective of this study was to evaluate a long-term (approximately 60-years) impact of agricultural reclamation on soil chemical properties as well as their SR features of a coastal saline marsh in Bohai Rim, northern China.

## Materials and Methods

### Study site

This study was conducted at a coastal permanent marsh and a cropland cultivated from the marsh in Qilihai (39°18′ N, 117°29′ E, elevation 2 m) of Bohai Rim, northern China. No specific permits were required for the described field studies in the research site. The field studies did not involve endangered or protected species. The study site is flat and low-lying. The region was characterized by a warm temperate semi-humid monsoon climate. Annual mean air temperature was 11.2 °C; total precipitation amount was 500–600 mm year^−1^; evapotranspiration was approximately 1900 mm year^−1^; frost-free period was 180–194 d; and sunshine duration hours were 2600–2800 h. Rainfall occurred synchronously with the heat, with high values in summer (June–August).

The permanent marsh was formed from the land-sea interactions since middle-late Holocene. The permanent marsh was mainly covered by reed (*Phragmites australis*) and generally had a water level of 0–40 cm above surface recently. Croplands were cultivated from the *P. australis* marshes over approximately 60 years. During the first ∼20 years (1950s–1960s), paddy was cultivated. Afterwards, the paddy lands converted to dry-lands mainly dominated by corn, cotton and sorghum in rotation. Organic (manure and straw) and chemical fertilizer applications during 1950s–1990s [23] were listed in Table 1. Since the new century, organic and chemical fertilizer applications [24] were presented in Table 2. The current water level of the dry-lands was about 80–100 cm below surface.

**Table 1:** Organic and chemical fertilizer applications during 1950s–1990s (source from the reference [23]).

	Organic fertilizer (manure and straw)		Chemical fertilizer	
	Application rate (m^3^ ha^−1^)	Proportion (%)	Application rate (kg ha^−1^)	Proportion (%)
1950s–1960s	30	93	53	7
1970s	40	61	600	39
1980s	20	40	690	60
1990s	20	29	1156	71

**Table 2:** Nitrogen and phosphorus inputs of organic and chemical fertilizer applications in 2007 (source from the reference [24]).

	Organic fertilizer (manure and straw)		Chemical fertilizer	
	Nitrogen (kg ha^−1^)	Phosphorus (kg ha^−1^)	Nitrogen (kg ha^−1^)	Phosphorus (kg ha^−1^)
2007	66	41	269	149

### Soil sampling and chemical analysis

The *P. australis* marsh and the cropland were adjacent (about 500 m) and had similar altitudes. Soil samples were randomly collected using a soil drill (Eijkelkamp, Netherlands) at three locations in the permanent marsh and cropland in May 2012, respectively. In both marsh and cropland, the distance between any two sampling locations was more than 15 m. In each sampling location, two adjacent soil columns (0–100 cm) were collected and then mixed into one column according to 7 corresponding horizons: 0–10, 10–20, 20–30, 30–50, 50–70, and 70–100 cm.

Soil samples were brought to laboratory and air-dried naturally. The air-dried soil samples were sieved with a 2 mm mesh screen to remove the roots and coarse fraction before chemical analysis. Dilute HCl (0.5 mol L^−1^) was used to remove soil inorganic carbon. Then deionized water was used to rinse off excess HCl and ensure the neutral pH of approximate 7. The contents of soil organic carbon (SOC) and total nitrogen (TN) were determined by an Elementar (Vario EL III, Elementar, Germany). The concentration of total phosphorus (TP) was determined by sulphuric and perchloric acids digestion and Mo-Sb Anti spectrophotometric method. Soil pH was measured with a pH meter (Star A 420C-01A, Thermo Orion, United States) using a soil:water ratio of 1∶2.5 (g g^−1^). Electrical conductivity (EC) was measured with a EC meter (Star A 420C-01A, Thermo Orion, United States) using a soil:water ratio of 1∶5 (g g^−1^). Soil major soluble cations (Na^+^, Ca^2+^, K^+^ and Mg^2+^) and anions (Cl^−^, SO_4_^2−^, HCO_3_^−^ and CO_3_^2−^) were measured using a soil:water ratio of 1∶5 (g g^−1^). Cation concentrations were determined by an Atomic Absorption Spectrophotometer (TAS-990, Beijing Purkinje General Instrument Co., Ltd., China). Concentrations of Cl^−^ and SO_4_^2−^ were determined by an Ion Chromatography (ICS-2100, Dionex Corporation, Sunnyvale, California, United States). Concentrations of CO_3_^2−^ and HCO_3_^−^ were measured using neutralization titration method using phenolphthalein and methyl orange.

### Data analysis

Sodium adsorption ratio (SAR), an easily measured property that gives information on the comparative concentrations of Na^+^, Ca^2+^, and Mg^2+^ in soil solutions, was calculated using equation (1) [25]. Exchangeable sodium percentage (ESP) was computed using equation (2) [26], [27]. Residual sodium carbonate (RSC) was estimated using equation (3) [28]. Comparisons of mean SOC, TN, TP, pH, EC and salinity between the marsh and cropland were analyzed using one-way ANOVA of SPSS 13.0 (SPSS Inc., Chicago, Illinois, United States). Figures were drawn using Origin 8.0 (OriginLabs Corporation, Northampton, Massachusetts, United States) and CorelDraw 9 (Corel Corporation, Canada).
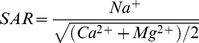
(1)

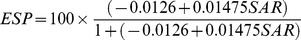
(2)

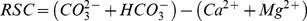
(3)


## Results

### Soil organic carbon, total nitrogen and phosphorus as well as their stratification ratios

**Figure 1:**
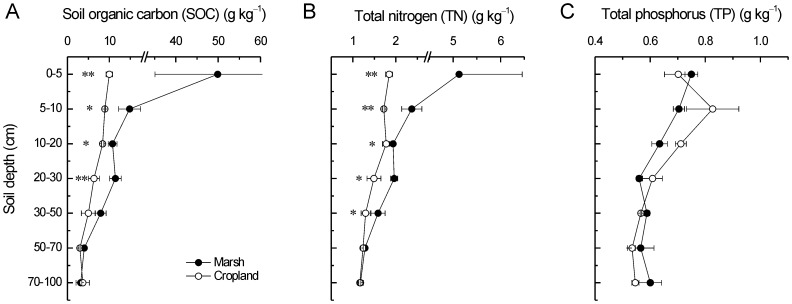
Long-term effects of reclamation on A) SOC, B) TN, and C) TP of the coastal marsh. * and ** indicate significant difference at *p*<0.05 and *p*<0.01 level, respectively.

**Figure 2:**
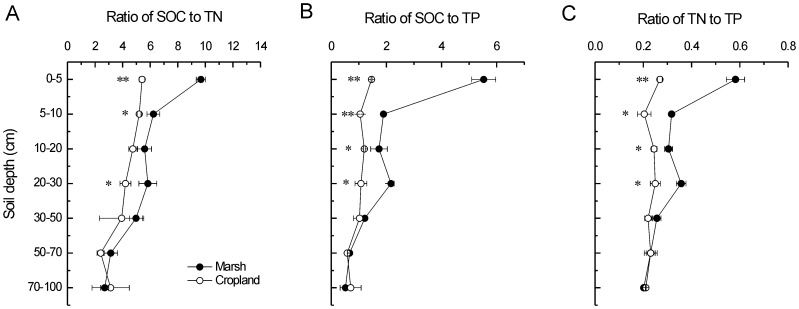
Long-term effects of reclamation on ratios of A) SOC/TN, B) SOC/TP, and C) TN/TP. * and ** represent significant difference at *p*<0.05 and *p*<0.01 level, respectively.

Long-term agricultural reclamation significantly reduced the concentrations of SOC at surface soil (0–30 cm) but not lower soil layers (30–100 cm) (Fig. 1A). The weighted density of SOC at surface soil (0–30 cm) decreased by 10.1 g kg^−1^ (−42.2%). Similarly, the contents of TN of upper soil layers (0–50 cm) but not deeper soil layers (50–100 cm) were also evidently decreased under long-term agricultural cultivation (Fig. 1B). The weighted density of TN at surface soil (0–50 cm) reduced by 0.7 g kg^−1^ (−31.8%). In contrast, there were no significant effects of long-term agricultural reclamation on the contents of TP at the whole soil profile (0–100 cm) (Fig. 1C). The ratios of SOC/TN, SOC/TP and TN/TP were all notably decreased after agricultural reclamation at surface soil (0–30 cm, except for 10–20 cm of SOC/TN) but not lower soil layers (30–100 cm) (Fig. 2).

**Figure 3:**
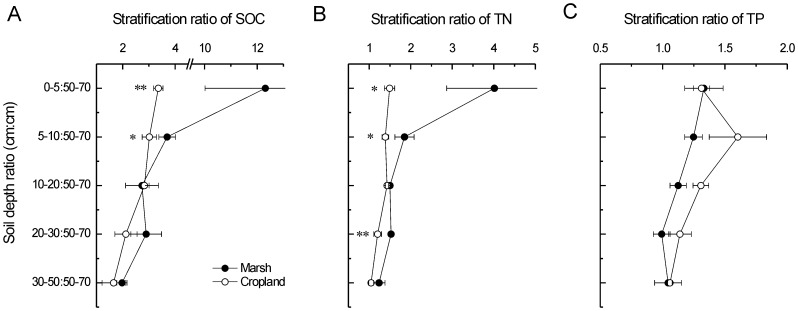
Long-term influences of reclamation on stratification ratios of A) SOC, B) TN, and C) TP. * and ** indicate significant difference at *p*<0.05 and *p*<0.01 level, respectively.

**Figure 4:**
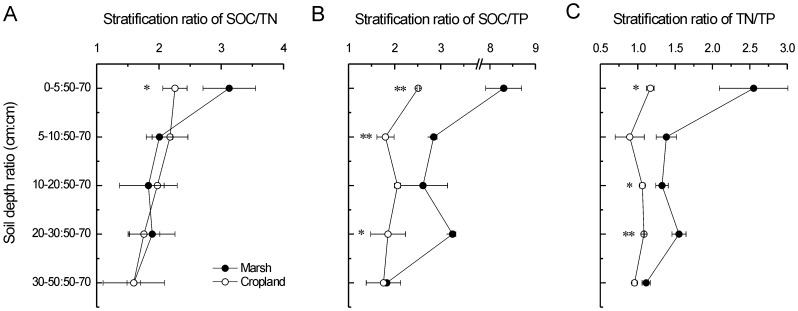
Long-term impacts of reclamation on stratification ratios of A) SOC/TN, B) SOC/TP, and C) TN/TP. * and ** indicate significant difference at *p*<0.05 and *p*<0.01 level, respectively.

Stratification ratios of 0–5 cm:50–70 cm and 5–10 cm:50–70 cm of SOC were 12.3 and 3.7 for the marsh and were 3.4 and 3.0 for the cropland (Fig. 3A). Stratification ratios of 0–5 cm:50–70 cm and 5–10 cm:50–70 cm of TN were 4.0 and 1.9 for the marsh and were 1.5 and 1.4 for the cropland (Fig. 3A). Compared to the marsh, SRs of SOC and TN of the cropland were both remarkably dropped, suggesting greater decreases of SOC and TN in the upper soil layers (Fig. 3A and B). In contrast, there was no significant impact of reclamation on the SR of TP (Fig. 3C). The SRs (0–5 cm:50–70 cm) of ratios of SOC/TN, SOC/TP and TN/TP were all significantly decreased after agricultural reclamation (Fig. 4).

### Soil EC, salinity and pH

**Figure 5:**
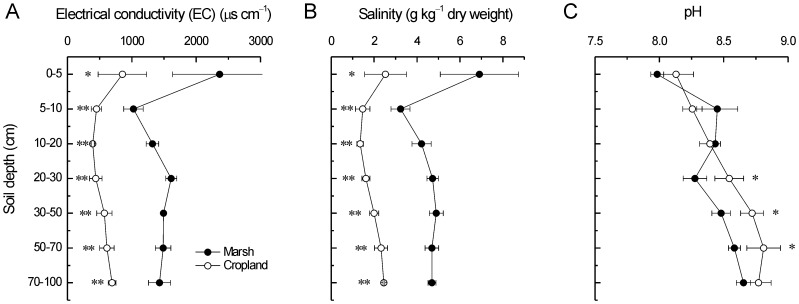
Long-term effects of reclamation on A) EC, B) salinity, and C) pH of coastal soils. * and ** indicate significant difference at *p*<0.05 and *p*<0.01 level, respectively.

Soil profile EC of the marsh ranged from 1026 to 2365 μs cm^−1^, with a weighted average of 1488 μs cm^−1^. Soil profile EC of the cropland varied from 392 to 854 μs cm^−1^, with a weighted average of 594 μs cm^−1^ (Fig. 5A). Soil profile salinity of the marsh was from 3.2 to 6.9 g kg^−1^ dry weight, with a weighted average of 4.7 g kg^−1^ dry weight. Soil profile salinity of the cropland ranged from 1.3 to 2.5 g kg^−1^ dry weight, with a weighted average of 2.1 g kg^−1^ dry weight (Fig. 5B). Thus, after long-term reclamation, EC and salinity of the whole soil profile (0–100 cm) decreased by 60.1% and 55.3%, respectively. In support of these results, concentrations of water-extractable ions showed great declines after reclamation. In contrast, soil pH (20–70 cm) significantly increased after reclamation.

### Compositions of cation and anion

**Figure 6:**
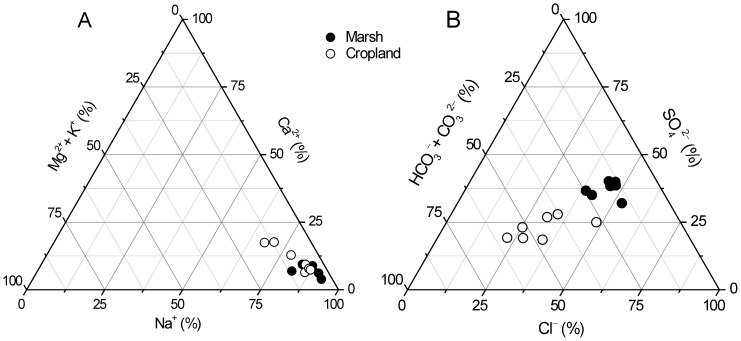
Long-term effects of agricultural cultivation on the compositions of A) cations and B) anions.

Even though the cations of marsh and cropland were both dominated by Na^+^, the proportion of Na^+^ (0–10 cm) was significantly decreased after reclamation (*p*<0.05) (Fig. 6A). However, the proportion of Ca^2+^ (0–10 cm) and Mg^2+^+K^+^ (5–20 cm) were significantly increased after reclamation (*p*<0.05) (Fig. 6A). Contrasted to cation, the composition of anion changed greater at 5–100 cm after reclamation, with significant decreases in Cl^−^ and SO_4_^2−^ and increases in HCO_3_^−^+CO_3_^2−^ (*p*<0.05) (Fig. 6B).

### Characteristics of alkalization

**Figure 7:**
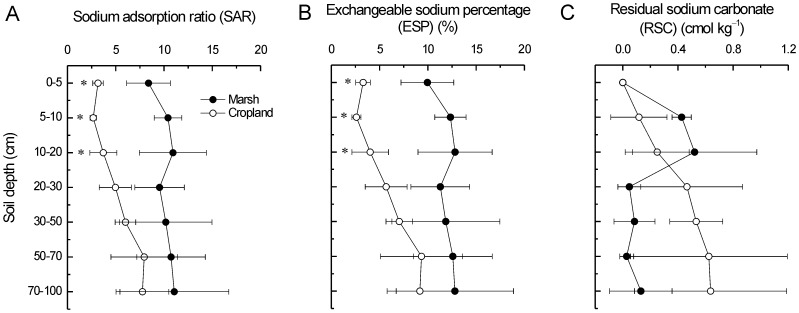
Long-term effects of reclamation on A) SAR, B) ESP, and C) RSC of the coastal marsh. ^*^ indicates significant difference at *p*<0.05.

The SAR and ESP in plow-layer (0–20 cm) significantly reduced after reclamation (*p*<0.05) (Fig. 7A and B). This result suggested a reduction in soil alkalization and sodium harm. However, there was no significant difference in RSC under long-term cultivation (Fig. 7C).

## Discussion

### SOC, TN and TP as well as their stratification ratios

The soil storages of SOC, TN and TP are counterbalances of inputs (e.g. productivity and fertilizer) and outputs (e.g. loss of mineralization and harvest) during a certain period. The rates of inputs and outputs in soils have been widely recognized to be sensitive to environmental changes (e.g. [29], [30], [31]) and human disturbance (e.g. [19], [20], [32]). Agricultural reclamation of wetland substantially altered the soil environmental conditions, thus should change soil stocks of SOC, TN and TP through breaking the balance of inputs and outputs. In our study, even though many organic (manure and straw) and chemical fertilizers were applied for a long-term time (Table 1 and Table 2), SOC and TN decreased strongly in surface soil after reclamation (Fig. 1), suggesting that the outputs were much larger than the inputs. Our result was in agreement with the results in the Yangtze Estuary [10] and the Hangzhou Bay of China [14]. Four reasons may explain the above results. First, the mineralization and accumulation of soil organic matter were both regulated by hydrologic conditions [12], [33]. Submerged soils of the marsh were under anaerobic conditions, which had lessened and incomplete decompositions of organic materials and decreased humifications of organic matter. In contrast, cropland soils lacked anaerobiosis and had high rates of decomposition due to the aerobic conditions. Compared to cropland soils, there were preferential accumulations of organic matter in submerged soils [12], [33]. Second, crop harvesting took lots of organic materials away from farmland ecosystem. Moreover, the net primary productivity of marshes is higher than cropland ecosystems [33]. In addition, decrease in soil salinity caused by agricultural reclamation might accelerate the mineralization of organic matters [16]. In our study, however, there was no significant change of SOC and TN in 50–100 cm soil under long-term reclamation (Fig. 1), indicating limited effects of reclamation on deeper soil horizons. This result was consistent with the results in the Sanjiang Plain [34], the Xingkai Lake [35] and the Hangzhou Bay of China [18]. Compared to SOC and TN, there was no significant variation in TP in whole soil profile after long-term reclamation (Fig. 1). However, Huang et al. [13] found significant decreases in TP caused by conversions of marshes to drylands. In marsh and cropland ecosystems, biogeochemical cycle of phosphorus is different from carbon and nitrogen. Phosphorus has poor mobility and is easy to be fixed in the soil. The input and output of phosphorus in the soil is mainly as fertilizer application and crop harvesting, respectively [36]. Thus, reclamation-induced impacts on TP may be explained by the balance of fertilizer application and crop harvesting.

Stratification ratio is a quantitative indicator to represent the responses of soil profile feature to anthropogenic disturbance [22], which was mainly applied in farmlands (e.g. [19], [20]). However, the application of SR is not common in costal wetlands. Our results showed that SRs (0–5 cm:50–70 cm and 5–10 cm:50–70 cm) of SOC and TN decreased remarkably after long-term agricultural reclamation. In support of this result, chemical properties of the surface soil were reported to be more sensitive to reclamation than that of the deeper layers in two coastal wetlands [4], [18]. These results are reasonable because the surface soils are the critical zones that receive intensive influences from anthropogenic activities.

### Salinity, ion composition and alkalization

Our results showed that soil salinity and EC across the whole profile (0–100 cm) dropped significantly after agricultural reclamation (Fig. 5 A and B). This result was in agreement with the results in the Yellow River Delta [13], the Jiangsu province [11], the Yangtze Estuary [10], the Hangzhou Bay of China [14] and the Biscay Bay of Spain [9]. A reliable interpretation for this result is the effective leaching of the soil exchangeable ions induced by dewatering after reclamation. However, each of the major ions (anions and cations) in soil solutions has some unique properties which affect its production and mobility [37]. For example, the production of bicarbonate (HCO_3_^−^), one of the most common anions, is regulated by soil CO_2_ pressure and pH. The sulfate (SO_4_^2−^) is involved in both biological and inorganic chemical reactions, whereas the chloride (Cl^−^) is relatively uninvolved in these reactions and thus has high mobility [37]. The cations are held by the negatively charged clay and organic matter particles in the soil through electrostatic forces. Therefore, ion compositions were proposed to be sensitive to disturbance due to the specific mobility of each ion. In support of this concept, we found that the proportion of Na^+^ (0–10 cm), Cl^−^ and SO_4_^2−^ (5–100 cm) decreased significantly after reclamation. However, the proportion of Ca^2+^ (0–10 cm), Mg^2+^+K^+^ (5–20 cm) and HCO_3_^−^+CO_3_^2−^ (5–100 cm) increased significantly after reclamation (Fig. 6). Knowing these reclamation-induced properties of the major ions, it is possible to protect and manage coastal saline soils of the wetlands and croplands as well as their ecosystem services. For example, reclamation-induced decreases in water table and soil salinity were beneficial to crop cultivation. High mobility of Na^+^ further reduced the harm of salt ions after reclamation. Even though overlying water or saturated soil are basic properties of coastal wetlands, reclamation-induced hydrologic alterations such as ditch drainage and diking result in decreases in salinity, which promotes plant activities and crop yields.

The SAR, ESP and RSC are three indicators of the degree of soil alkalization. The SAR of a soil extract takes into consideration that the adverse effect of Na^+^ is moderated by the presence of Ca^2+^ and Mg^2+^. When the SAR rises above 12 to 15, serious physical soil problems arise and plants have difficulty absorbing water [38]. In our study, all SARs of the marsh and cropland were below 12. Our results showed that agricultural reclamation significantly decreased the SAR and ESP but not RSC in the plow-layer (0–20 cm), suggesting a reduction in Na^+^ harm (Fig. 7). These results were consistent with those in the Cape Cod, Massachusetts of USA [8] and the Hangzhou Bay of China [14]. Moreover, significant decreases of SAR and ESP in the upper soil horizons further supported the rapid desalinization.

## Conclusions

In coastal marshes of northern China, long-term agricultural reclamation significantly reduced SOC and TN at surface soil (0–30 cm) as well as their SRs (0–5 cm:50–70 cm and 5–10 cm:50–70 cm). However, there was no significant change in TP as well as its SRs. Reclamation notably decreased ratios of SOC/TN, SOC/TP and TN/TP at surface soil (0–30 cm) as well as their SRs (0–5 cm:50–70 cm). A significant desalinization of whole soil profile occurred during cultivation. Soil pH (20–70 cm) markedly increased as an impact of reclamation. Compositions of cations (0–10 cm) and anions (5–100 cm) changed mainly as decreases in proportion of Na^+^, Cl^−^ and SO_4_^2−^. Furthermore, reclamation significantly reduced the sodium adsorption ratio and exchangeable sodium percentage in plow-layer (0–20 cm) but not residual sodium carbonate, suggesting a reduction in sodium harm.
